# The Role of Perspective-Taking on Ability to Recognize Fear

**DOI:** 10.3844/crpsp.2015.22.30

**Published:** 2016-01-12

**Authors:** Andrea Trubanova, Inyoung Kim, Marika C. Coffman, Martha Ann Bell, J. Anthony Richey, Stephen M. LaConte, Denis Gracanin, Susan W. White

**Affiliations:** 1 Department of Psychology, Virginia Polytechnic Institute and State University, Blacksburg, Virginia, USA; 2 Department of Statistics, Virginia Polytechnic Institute and State University, Blacksburg, Virginia, USA; 3 Department of Biomedical Engineering and Sciences, Virginia Tech Carilion Research Institute, Roanoke, Virginia, USA; 4 Department of Computer Science, Virginia Polytechnic Institute and State University, Blacksburg, Virginia, USA

**Keywords:** Perspective-Taking, Emotion Recognition, Fear, Empathy

## Abstract

Impairment in the ability to detect certain emotions, such as fear, is linked to multiple disorders and follows a pattern of inter-individual variability and intra-individual stability over time. Deficits in fear recognition are often related to social and interpersonal difficulties but the mechanisms by which this processing deficit might occur are not well understood. One potential mechanism through which impaired fear detection may influence social competency is through diminished perspective-taking, the ability to perceive and consider the point of view of another individual. In the current study, we hypothesized that intra-individual variability in the accuracy of facial emotion recognition is linked to perspective-taking abilities in a well-characterized, non-clinical adult sample. Results indicated that the ability to accurately detect fear in the faces of others was positively correlated with perspective-taking, consistent with initial hypotheses. This relationship appeared to be unique to recognition of fear, as perspective-taking was not significantly associated with recognition of the other basic emotions. Results from this study represent an initial step towards establishing a potential mechanism between some processes of FER and perspective-taking difficulties. It is important to establish the relationship between these processes in a non-clinical adult sample so that we can consider the possibility of a developmental or pathological influence of impoverished perspective-taking on fear perception.

## Introduction

Facial emotion recognition, the ability to accurately identify and interpret facial expressions emerges early in life and is crucial for effective social interaction. Impaired facial emotion recognition has been observed in neurological and psychiatric disorders (Donno *et al*., 2010). Specifically, impaired ability to detect another person’s fear, a distressing emotion aroused by threat of impending or possible danger, has been linked to many types of psychopathology (Gross and Jazaieri, 2014). However, the association between ability to recognize facial expressions and social functioning has not been well studied in healthy, non-psychiatric sample. Outside of psychopathology, it is important to see if and how fear recognition differs from recognition of other emotions in non-clinical individuals. Although the mechanisms through which impoverished fear detection might cause interpersonal difficulties are not well understood, one potential process is impaired perspective-taking, an ability to consider the world from other viewpoints and “allows an individual to anticipate the behavior and reactions of others” (Davis, 1983, p. 115). Accuracy of facial expression recognition may be a primary determinant of observed individual differences in processes such as interpersonal insight and perspective-taking. Thus, the goal of the current study was to examine the association between perspective-taking and emotion recognition in a well characterized, non-clinical adult sample.

The development of emotion recognition appears to be important for normative interpersonal functioning, as the ability to recognize and label emotion expressions predicts positive social interactions, as well as academic competence in young children (Izard *et al*., 2001). Conversely, difficulties with emotion recognition have been implicated in many forms of psychopathology (Gross and Jazaieri, 2014) including early-onset conduct disorder (Fairchild *et al*., 2009), depression (Dalili *et al*., 2014), Autism Spectrum Disorder (ASD; Uljarevic and Hamilton, 2013) and psychopathic traits (Dadds *et al*., 2008). Although it is possible that an individual can have broadly impaired recognition across a range of emotions, there is considerable evidence that impairment in recognition of specific emotions, fear in particular, is linked to psychopathology.

Collectively, extant research suggests that impaired fear recognition may be a stronger predictor of psychopathology than impairment in the recognition of other emotions (Demirel *et al*., 2014; Montagne *et al*., 2005). Impaired recognition of fear has been found to be associated with criminal behaviors among people with schizophrenia (Weiss *et al*., 2006) and there is a robust association between inability to perceive fear via facial cues, antisocial behavior and a lack of empathy (Blair *et al*., 2001; Montagne *et al*., 2005; Stevens *et al*., 2001). Impairment in the ability to perceive fear has also been documented in bipolar disorder (Demirel *et al*., 2014), ASD (Humphreys *et al*., 2007) and ADHD (Aspan *et al*., 2014). Collectively, impairment in fear recognition has been observed across multiple disorders that share difficulties in social interaction. In light of the social and interpersonal difficulties that may result from impaired fear recognition (Corden *et al*., 2008; Skuse, 2003), an improved understanding of this process may facilitate the development of new intervention concepts that are applicable across a range of psychiatric disorders characterized by social disability. One such potential mechanism is perspective-taking, a multi-faceted construct often impaired across disorders. Both perspective-taking and facial emotion recognition are necessary aspects for successful social interactions.

Perspective-taking refers to the ability to perceive, appreciate and consider the perspective, or point of view, of another individual. It has long been recognized as a critical aspect for successful social interaction. Studies in clinical and non-clinical samples have shown a positive association between perspective-taking and interpersonal skills (Gerace *et al*., 2013; Marsh *et al*., 1981), highlighting the importance of perspective-taking in everyday interactions. Many of the populations struggling with decreased perspective-taking also show deficits in emotion recognition. For example, a study by Seidel *et al*. (2013) found reduced accuracy in violent offenders compared to non-psychiatric controls in emotion recognition and an association between a high number of violent assaults and decreased accuracy in perspective-taking for angry scenes. In addition, deficits in perspective-taking have been implicated in a subset of individuals with conduct disorder who show low callous-unemotional traits (Anastassiou-Hadjicharalambous and Warden, 2008) and in individuals with ASD (Baron-Cohen *et al*., 2000), both of which are clinical populations characterized by social problems and who often show deficits in emotion recognition abilities.

Emotion recognition emerges early in life, with newborns being able to discriminate among some facial expressions (Field *et al*., 1982). The early roots of perspective-taking, on the other hand, are not present until after the first year (Sodian *et al*., 2007), with higher-level perspective-taking abilities emerging around 4 or 5 years of age (Frick *et al*., 2014). As such, it may be that weakened recognition of fear precedes impaired perspective-taking developmentally. Although these processes are conceptually and developmentally distinct, linkages between the two have been established by intervention studies showing that treatments addressing social cognition impairments, such as diminished perspective-taking, often affect emotion recognition in tandem. For example, a study by Gibson *et al*. (2014) showed that administration of oxytocin to adults with a schizophrenia diagnosis not only improved perspective-taking, but also improved fear recognition. Nantel-Vivier *et al*. (2011) found that boys with elevated physical aggression, when given tryptophan, improved in both their perspective-taking and ability to distinguish facial expression of fear. It is important to establish a relationship between these concepts outside of a treatment study. Even highly focused treatments often influence other, non-targeted processes indirectly (Borkovec *et al*., 1995), suggesting that observation of change in a secondary construct (e.g., emotion recognition) does not establish its relationship to the target outcome (e.g., perspective-taking). In order to identify candidate mechanisms for prevention or intervention efforts in clinical samples, links between the proposed mechanisms and behaviors of interest must first be established. Secondly, in order to understand the relationship between processes across disorders as well as outside of psychopathology (i.e., as continuously distributed individual human differences), it is important to explore them concurrently, in a non-clinical population.

Based on prior research showing that emotion recognition temporally precedes perspective-taking developmentally and that altering perspective-taking can secondarily alter fear recognition ability, we propose that perspective-taking and impaired fear recognition ability are related processes. In the present study, we sought to examine the connection between recognition of fear, relative to the other basic emotions and perspective-taking. Specifically, we hypothesized that there is a positive association between perspective-taking and accuracy for recognition of fear in non-clinical, adult sample.

## Method

### Participants

A sample of 20 non-clinical adult males enrolled into a larger study evaluating emotion recognition accuracy through electroencephalography (EEG) and functional Magnetic Resonance Imaging (fMRI). Participants were recruited through flyers posted in areas near the University campus (e.g., coffee shops and on-campus buildings) as well as through the Department of Psychology website and through the University Graduate School. Interested participants were directed to contact the study investigator by phone or email. Only the behavioral data from the project are analyzed and reported in this study. Table 1 displays the participant characteristics. Inclusion criteria required participants to be: (1) Male; (2) between ages 18 to 28, inclusive; (3) of at least average cognitive ability (IQ equal to or above 80), as confirmed by Wechsler Abbreviated Scale of Intelligence, 2^nd^ edition (WASI-II; Wechsler, 2011); (4) capable of undergoing imaging (no MRI contraindications such as metal in body); (5) healthy with no known genetic, medical, or neurological conditions; (6) ambulatory with no known, uncorrected sensory deficits; (7) psychologically healthy (no diagnosed mental health conditions such as depression and anxiety, as confirmed by a clinical interview (Anxiety Disorders Interview Schedule for DSM-5 [ADIS-5, Silverman and Albano, 1996]); and (8) using no psychotropic medications. Females were excluded given the nature of the broader study with the focus on neural response to social stimuli, which has been shown to differ between sexes, reflecting influential differences in social information processing between males and females (Coffman *et al*., 2015). In addition, females were excluded given the power needed to explore sex differences within the study. A young adult sample was utilized because the focus of the broader study was to design a facial emotion recognition intervention developed for use by adults. Eight additional participants completed the assessment portion of the study but were excluded due to moving out of state (*n* = 1), meeting criteria for mental health condition (*n*= 4), consistent and current drug use (*n* = 2), or not attending subsequent scheduled sessions (*n* = 1).

### Procedure

Interested participants completed an initial phone screen to answer preliminary eligibility questions, followed by one in-lab session to confirm study eligibility (e.g., clinical interview, cognitive assessment). If they met all inclusion criteria, participants were then scheduled for two experimental visits (the EEG and fMRI portions of the study completed for purposes of a larger study). The order of the two visits was counterbalanced so that half of the participants completed the EEG session first and the other half of the participants completed the fMRI session first. The two experimental visits were scheduled to occur within 7 to 21 days of each other. During these visits, participants completed the emotion recognition task and the remaining behavioral measures and questionnaires. The two sessions were otherwise equal in the task that participants completed, described below. All participants received $25 for each session, for a total of $75 for completion of all three in-person visits.

### Perspective-Taking

Perspective-taking was measured with the Interpersonal Reactivity Index (IRI; Davis, 1980), a 28-item self-report scale of empathic ability, such as ability to take another’s perspective. Items are answered on a 5-point Likert scale ranging from “does not describe me well” to “describes me very well”. The total IRI score is comprised of four subscales: Perspective-taking, Fantasy, Empathic Concern and Personal Distress. Internal reliability ranges from alpha = 0.70 to 0.78 for the four subscales, indicating that the measure appears to reliably tap four separate empathy factors (Davis, 1980). The Perspective-taking subscale measures the tendency of the participant to take the point of view of others (e.g., “When I am upset at someone, I usually try to ‘put myself in his shoes’ for a while”). Only the Perspective-taking subscale was examined for this study due to the specific focus on perspective-taking, a cognitive empathy, which is often dissociated from the emotional empathy in clinical populations (e.g., Dziobek *et al*., 2008).

### Emotion Recognition

Emotion Recognition was assessed through a behavioral task completed during fMRI and EEG data collection, for purposes or a larger study. The images were selected from the Cohn-Kanade image database (http://www.pitt.edu/~emotion/ck-spread.htm), due to a number of factors that ensure consistency among the videos, including: direct gaze of actors, size and distance of the faces from the camera and plain backgrounds. Further, all images have been Facial Action Coded and Emotion Facial Action coded, meeting the standards for portraying prototypical emotional expressions (Kanade *et al*., 2000). Lucey *et al*. (2010) evaluated the validity of the emotion labels and found acceptable machine-based agreement between presented and detected emotion. For this study, the images from the Cohn-Kanade image set were individually reviewed and consensus rated by two graduate research assistants for authenticity of the emotion. Selected face images portrayed six basic emotions (i.e., anger, fear, disgust, surprise, happiness, sadness), such that there were 10 image sets for each emotion. The number of times an individual actor was portrayed was limited to three presentations, in order to reduce participant familiarity with the actor. Only male faces were selected, due to the all-male participant sample. The facial emotions were shown through a series of still images (which were compiled into a dynamic video, as described below). The final group of 60 videos was then split into 2 sets (for two tasks), each comprised of 30 emotion videos, so that no videos were repeated within a visit. Only one task was analyzed for this study and therefore the data includes information from 30 stimuli repeated during the two visits. Each actor was shown no more than 2 times per condition and each emotion was shown 5 times per task. The videos were created using custom MATLAB code that interpolated the space between the still images to make the videos appear smoother. On average, the videos were 20.57 sec (*SD* = 7.26) in length across different emotions and did not vary in length across emotional conditions. The presentation of stimuli was randomized for each participant. The participant was asked to respond with a button press as soon as he recognized the emotion. After the completion of the video, the participant was asked to select the portrayed emotion from a list of six presented emotions.

## Results

There was no difference between EEG and fMRI sessions in terms of accuracy in the emotion recognition paradigm for any of the emotions [all *F*(1, 18) <1.92, all *p*>0.18] or in perspective-taking scores [*F*(1, 18) = 1.80, *p* = 0.20]. Data were therefore combined across the two sessions.

### Emotion Recognition and Perspective-Taking Scores

**Fig. 1:**
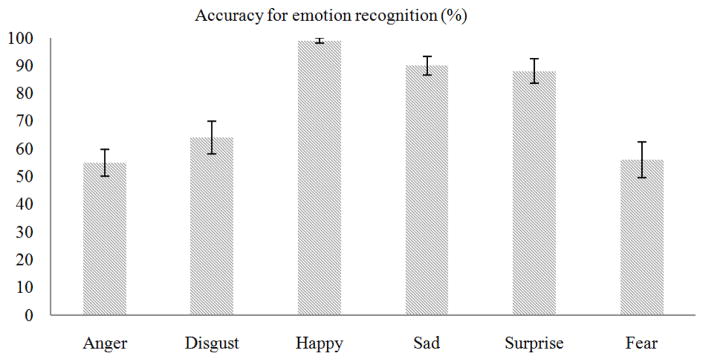
Descriptive statistics for emotion recognition reported as percentage of accurately identified emotions for each emotion type

Scores on the perspective-taking subscale of the IRI ranged from 10 to 26 (*M* = 19.60, *SD* = 4.08). Average accuracy for recognition of emotion, collapsed across emotions, was 75.33% (*SD* = 8.54). See Fig. 1 for the average accuracy for each of the six emotions. Table 2 portrays the misattribution matrix illustrating the emotion participants chose when they did not select the correct emotion. There was a difference in accuracy among emotions as determined by one-way ANOVA, *F*(5,114) = 16.898, *p* < 0.001. Post hoc comparisons using the Tukey HSD test indicated higher accuracy for happy (*M* = 99.00, *SD* = 4.47), sad (*M* = 90.00, *SD* = 15.22) and surprise (*M* = 88.00, *SD* = 19.89) compared to anger (*M* = 55.00, *SD* = 21.40), disgust (*M* = 64.00, *SD* = 26.44) and fear (*M* = 56.00, *SD* = 28.73). However, accuracy for anger, disgust and fear did not differ from each other (all *p*’s>0.75) and accuracy for happy, sad and surprise emotions were not different from each other (all *p*’s>0.56). When incorrectly identified, emotions were not reciprocally or equally misidentified (e.g., surprise as fear and fear as surprise). Rather, as shown in Table 2, surprise was most often confused with fear, fear was most often confused with disgust and disgust was most often confused with anger.

### Relations between Fear Recognition and Perspective-Taking

Statistical analyses for associations between fear recognition and perspective-taking were performed using R (RDCT, 2015). Due to the small sample size and, therefore, uncertain underlying distribution of the data, the data distribution was first checked for satisfaction of the normality assumption using the Shapiro-Wilk Test (Oztuna *et al*., 2006) and found to be violated. Given the small sample size and non-normal distribution, a bootstrapping nonparametric approach (Efron and Tibshirani, 1993) was used to test the model (i.e., accuracy for fearful stimuli = β0 + β1 (perspective-taking scale on empathy total) + δ, where β0 and β1 are unknown model parameters and δ is the measurement error). We estimated unknown parameters and calculated the 95% confidence interval of β1 based on 1,000 bootstrapping procedure. Results show that the 95% confidence interval (0.0182, 6.665) does not contain zero, indicating a positive relationship between perspective-taking and accuracy in fear recognition. A Spearman correlation (*R* = 0.422; *p* = 0.036), conducted as a secondary analysis, further supports the finding. Perspective-taking did not correlate with any emotions (all *p*-values > 0.165) except with fear. See Table 3 and 4 for complete Bootstrapping and Spearman correlation results.

## Discussion

In a non-clinical adult sample, the ability to accurately detect fear in the faces of others was found to be positively correlated with perspective-taking abilities. This association is unique to recognition of fear, as perspective-taking was not significantly associated with recognition of the other basic emotions. Prior research has suggested a unique role for impaired fear recognition, more so than broad emotion recognition deficits, in multiple forms of psychopathology (e.g., Montagne *et al*., 2005; Weiss *et al*., 2006). These results extend these findings within a non-clinical sample and provide preliminary support for a possible mechanistic relationship between impaired fear recognition and impaired perspective-taking.

Results of this study suggest that perspective-taking is linked to fear recognition difficulties. Fear recognition ability, we assert, is a fairly stable individual difference that may predict perspective-taking as well as social functioning among non-psychiatric adults, as well as be predictive of psychopathology. What is unique about fear in terms of emotion recognition and perspective-taking? When considering the possibility of emotion-specific impairments, it is important to examine the potential role of task difficulty. We found that emotion recognition accuracy in non-clinical adult males was highest for happiness, followed by sadness and surprise. Accuracy was lowest for anger and fear. These results are consistent with prior cross-cultural studies showing that recognition scores are highest for happiness and lowest for fear (Biehl *et al*., 1997). In addition, fear was most often misrecognized as disgust, followed by sadness, consistent with other findings (Kohler *et al*., 2014). Accuracy rates for fear expression are relatively low for non-clinical and clinical populations alike. Fear, however, is not more difficult to discriminate from neutral expression than are the other emotions (Adolphs, 2002) and, in the present study, we observed similarly low accuracy for other emotions, including anger and disgust.

Fear may be considered a valuable social signal, contributing to its association with perspective-taking. For example, fearful expression, in addition to sad facial emotion, is thought to serve as a social cue that conditions a person to avoid engaging in antisocial behaviors that elicit such expression (Marsh and Blair, 2008). Impairment in fear recognition could prevent a person (child or adult) from learning valuable social lessons, including appreciating other’s perspectives. Impaired perspective-taking therefore could result from the impairment in recognizing facial expressions that are important in social interactions. Results from this study support this premise, given no other emotions showed a significant association with self-reported perspective-taking. Additionally, research has shown that similar past experiences, as well as the current mood, are important in perspective-taking abilities (Gerace *et al*., 2015). Negative emotions, such as fear, might be harder to elicit for some individuals which might compromise their ability to take other person’s point of view.

Given the relationship between perspective-taking and fear recognition, we suggest that diminished perspective-taking may be an intervening process (or mechanism), through which impaired fear recognition ability contributes to social problems. It is also possible, however, that another factor (or factors), not considered in this study, explains the relationship found between fear recognition and perspective-taking. For example, motivation could be the mechanism contributing to the relationship, as individuals with deficits in recognition of fearful expressions could be less motivated to attend to and incorporate social (e.g., facial) cues which might results in lower perspective-taking in terms of lower motivation to show empathy. While this study shows the relationship between the processes, it does not exclude a mechanism not explored in this study.

Results should be evaluated in light of study limitations. First, the sample is small and homogeneous, comprised of twenty adult males. It is important to investigate the generalizability of the results to a broader population (i.e., females, children). Second, perspective-taking in this study was measured with a single, self-report trait measure. This limits the interpretation of the findings to a trait measure of empathy, or a consistent pattern of participant’s behaviors and abilities, as opposed to their current state of empathy which might differ based on the situation. Future studies would benefit from exploring different aspects of perspective-taking through a more comprehensive account of perspective-taking abilities. Although we showed an association between impairment in facial emotion recognition of fear and deficits in perspective-taking, future research needs to temporally establish the mechanism by which impairments occur and how this knowledge can be used to help individuals who show deficits in recognition of fearful facial expressions.

## Conclusion

Impairments in perspective-taking and fear recognition have long been studied in numerous forms of psychopathology. This study shows a positive relationship between the two concepts. These results suggest that impaired perspective-taking may be mechanistically or developmentally linked to impaired ability to recognize facial emotion of fear. These findings represent an initial step toward establishing a potential mechanism for perspective-taking difficulties by showing a clear association between perspective-taking ability and deficits in fear recognition in a non-clinical sample. Given that facial recognition emerges before perspective-taking skills, it is likely that emotion recognition deficits lead to the difficulty with perspective-taking and not the other way around.

Transdiagnostic models allow for identification of fundamental processes underlying psychopathology. Such processes are believed to operate dimensionally, not limited to a specific setting or disorder and as such be present in non-clinical individuals without psychopathology as well. However, the mechanism(s) through which a risk process comes to manifest as problematic are not well understood (Nolen-Hoeksema and Watkins, 2011). Our study raises several questions related to the non-clinical subject’s ability to recognize fear expression from dynamic expressions of emotion, given the relative low accuracy of identifying fear compared to other emotions. These results have potential implications for understanding widespread processes that can affect non-clinical functioning as well as assessment and treatment approaches of disordered behavior. A richer understanding of the proximal effects of facial emotion recognition differences, specifically with respect to fear in others, in typical adults may inform clinical research, as it can help to identify possible modifiable mechanisms which can be targeted therapeutically.

## Figures and Tables

**Table 1:** Demographics and descriptive data (*n* = 20)

	*n* (%)	M (*SD*)
Race		
Caucasian	9 (45%)	
African American	4 (20%)	
Asian	4 (20%)	
Mixed	3 (15%)	
Education Level		
High School Diploma	0 (0%)	
Some College	7 (35%)	
College Diploma	8 (40%)	
Graduate School	5 (25%)	
Age		23.4 (2.87)
IQ		110 (8.64)

**Table 2:** Misattribution matrix from emotion recognition task

Chosen Emotion	Presented emotion					
	Anger	Disgust	Sad	Fear	Surprise	Happy
Anger	55	34	1	1	1	1
Disgust	17	64	3	32	0	0
Sad	15	2	90	5	0	0
Fear	11	0	1	56	11	0
Surprise	1	0	5	4	88	0
Happy	0	0	0	2	0	99

**Table 3:** Summary of results from bootstrapping approach

	Mean	Median	SD	Boot CI 2.5%^a^	Boot CI 97.5% a
Anger	−0.520	−0.678	1.178	−2.275	2.322
Disgust	1.115	1.182	1.860	−3.093	4.564
Fear	2.861	2.705	1.7190	0.0182	6.665*
Happy	2.572e-01	1.996e-01	2.824e-01	−1.159e-15	9.529e-01
Sad	−0.546	−0.602	0.968	−2.337	1.531
Surprise	−1.371	−1.244	1.220	−4.048	0.495
Total	0.277	0.197	0.489	−0.479	1.465

a Boot CI 2.5% and Boot CI 97.5% represent the lower and upper bounds of 95% confidence interval based on 1,000 bootstrapping procedure;

* Indicates significant correlation where 95% CI contains zero

**Table 4:** Summary of results from Spearman correlation test

	Correlation Rho	*p*-value
Anger	−0.087	0.362
Disgust	0.120	0.313
Fear	0.422	0.036*
Happy	0.237	0.164
Sad	−0.222	0.181
Surprise	−0.188	0.221
Total	0.029	0.452

* indicates significant correlation
